# Assessing turbine passage effects on internal fish injury and delayed mortality using X-ray imaging

**DOI:** 10.7717/peerj.9977

**Published:** 2020-09-16

**Authors:** Melanie Mueller, Katharina Sternecker, Stefan Milz, Juergen Geist

**Affiliations:** 1 Aquatic Systems Biology Unit, Department of Ecology and Ecosystem Management, Technical University of Munich, Freising, Bavaria, Germany; 2 Institute of Anatomy, Faculty of Medicine, LMU Munich, Munich, Bavaria, Germany

**Keywords:** Animal welfare, Fish conservation, Fish mortality, Fish passage, Fish radiographic anatomy, Fish health

## Abstract

Knowledge on the extent and mechanisms of fish damage caused by hydropower facilities is important for the conservation of fish populations. Herein, we assessed the effects of hydropower turbine passage on internal fish injuries using X-ray technology. A total of 902 specimens from seven native European fish species were screened for 36 types of internal injuries and 86 external injuries evaluated with a previously published protocol. The applied systematic visual evaluation of X-ray images successfully detected skeletal injuries, swim bladder anomalies, emphysema, free intraperitoneal gas and hemorrhages. Injuries related to handling and to impacts of different parts of the hydropower structure could be clearly distinguished applying multivariate statistics and the data often explained delayed mortality within 96 h after turbine passage. The internal injuries could clearly be assigned to specific physical impacts resulting from turbine passage such as swim bladder rupture due to abrupt pressure change or fractures of skeletal parts due to blade-strike, fluid shear or severe turbulence. Generally, internal injuries were rarely depicted by external evaluation. For example, 29% of individuals with vertebral fractures did not present externally visible signs of severe injury. A combination of the external and internal injury evaluation allows quantifying and comparing fish injuries across sites, and can help to identify the technologies and operational procedures which minimize harm to fish in the context of assessing hydropower-related fish injuries as well as in assessing fish welfare.

## Introduction

Animal and fish welfare have recently received increased public and scientific interest (Stevens et al., 2017; Gordon et al., 2018; Saraiva & Arechavala-Lopez, 2019), including changes in policy and environmental legislation. Welfare issues not only apply to farmed fish, ornamental/pet fish, commercial- or recreational-fishing, but also to wild fish affected by anthropogenic changes to the environment (Huntingford et al., 2006), for example, including fish passage at hydropower plants or pumping stations. The threat to fish welfare, arising from an increasing use of hydropower turbines worldwide, is currently intensively discussed and studied (Baumgartner et al., 2017; Botelho et al., 2017; Amaral et al., 2018; Havn et al., 2018; Alves et al., 2019; Renardy et al., 2020).

Injury assessments on fish after hydropower turbine passage often rely on a general visual “expert judgement” or modeling results (Saylor, Fortner & Bevelhimer, 2019), determination of percentages of dead fish or on externally visible injuries. Although extensive evaluations of fish injury have been conducted, detailed procedures for evaluations of fish damage, considering type, severity and location of the injuries, have not been published until recently (Mueller, Pander & Geist, 2017). Immediate and delayed fish mortality has commonly been observed after passage through river infrastructure and obstacles without any externally visible signs of severe injury (Mueller, Pander & Geist, 2017; Bierschenk et al., 2019). These fish may have sustained internal injuries that did not present external indications of the physical stressors they experienced during turbine or pump passage, such as injuries of the vertebral column or swim bladder rupture (Brown et al., 2013; Colotelo et al., 2018; Pflugrath, Boys & Cathers, 2018).

Knowledge on internal injuries could help identify the physical causes of mortality and improve turbines towards a design that minimizes such impacts. During hydropower passage, fish experience massive physical forces, such as rapid decompression, collisions with high *g*-force, or fluid shear, which may not only damage the fish body externally, but also or exclusively internal viscera (Deng et al., 2005; Saylor, Fortner & Bevelhimer, 2019; Pflugrath et al., 2020). Fish that show externally visible injuries like bruises, scale loss, hemorrhages or disorientation, likely also suffer from internal injuries which often can be more directly related to their physical cause than external injuries. For instance, damage of the skeleton potentially may result from shear or collision, or swim bladder overinflation or rupture from decompression. The susceptibility to different types of internal injuries is supposed to be species-specific due to anatomical differences. For example, species differ in swim bladder morphology, with physoclists (e.g., Percids) lacking a connection of the swim bladder to the esophagus and therefore being more prone to swim bladder rupture than physostome species (e.g., Salmonids, Cyprinids). Also, the thickness of the swim bladder wall and the presence of two or more chambers may influence the ability to compensate for rapid pressure changes (Silva et al., 2018). Body size and shape can also strongly influence internal fish injuries, particularly skeletal injuries due to differences in collision risk.

Different diagnostic methods to detect internal fish injury are available, for example, including necropsy, X-ray imaging, Computer Tomography (CT) or Magnetic Resonance Imaging (MRI, e.g., Rogers et al., 2008 and Roach, Hall & Broadhurst, 2011). The most commonly used method is probably necropsy (McMichael, 1993; Boys et al., 2016; Beirão et al., 2018; Pflugrath et al., 2019), despite being a highly invasive procedure, due to its low costs and the possibility to determine injuries in specific organs (e.g., heart, spleen, liver). Diagnostic imaging methods bear minimum risk of preparation artifacts, can also be applied to live fish and deliver high resolution images of the interior of the fish body (Love & Lewbart, 1997; Wildgoose, 2003; Neues & Epple, 2008). Whilst CT and MRI are hardly accessible for animal studies and very expensive (e.g., costs for a CT of one fish is currently around 600 ₠), the necessary equipment and knowledge for conventional radiography (X-ray) is much more broadly available (Wildgoose, 2003).

The X-ray technique is particularly suitable to evaluate skeletal injuries and swim bladder anomalities (Love & Lewbart, 1997; Bakal et al., 1998), which are difficult to examine using necropsy since the swim bladder is easily damaged in some species and not easily accessible in many species (e.g., Siluriformes). Moreover, the skeleton is not easy to separate from the surrounding tissue using necropsy. In the context of hydropower-related effects on fish welfare, it is exactly these types of injuries that are of special interest as they directly relate to physical impacts such as pressure change (barotrauma), fluid shear or blade strike and collision with other parts of the power plant such as the fish exclusion screen. Therefore, X-ray imaging can be an ideal complementary method to necropsy to gain more comprehensive insights on internal injuries or a substitute where an assessment of live fish is to be preferred. X-ray imaging has already been used in scientific studies on hydropower-related fish injury (Brown et al., 2013; Geist et al., 2013) and even in technical monitoring studies by private consultancies (Schneider, Hübner & Korte, 2012), indicating practical feasibility of the method. On the other hand, interpretation was so far rather descriptive or restricted to very specific details and did not follow any standardized protocol which would allow comparability among studies.

Besides the effects of hydropower turbine passage, internal fish anatomy can be influenced by other stressors such as genetic constitution, exposure to pesticides, sport fishing related injuries, or malnutrition in aquaculture (Wildgoose, 2003; Gorman, Tredwell & Breden, 2007; Noble et al., 2012; Bowersox et al., 2016). An assessment of internal injuries could therefore also contribute to a more comprehensive evaluation of fish health and welfare in other contexts such as wild fish populations, aquaculture or pet fish trade.

Herein, we assess the effects of hydropower turbine passage on internal injuries of fish based on X-ray imaging. We investigated three main questions using an experiment at a hydropower plant with a Kaplan turbine: (i) Is a visual screening of lateral X-ray views for swim bladder anomalies as well as skeletal and soft tissue injuries (fractures, deformities, emphysema, fluid accumulations) sufficient to distinguish internal fish injuries related to turbine passage from handling effects and naturally occurring anomalies? (ii) Can immediate and delayed mortality be assigned to specific internal injuries detected by the X-ray method? (iii) How are externally visible injuries and internal injuries related? (iv) Do types and intensities of internal injuries differ among fishes with morphological differences in body shape and swim bladder type?

## Materials and Methods

The experiment was approved for appropriate animal care and use according to European laws (European Parliament, 2010) as well as national standards and guidelines (Adam, Schürmann & Schwevers, 2013) by the Bavarian Government (permit number ROB-55.2-2532.Vet_02-15-31). Application documents received ethical approval by the TUM animal welfare officer and the Ethical Commission of the Bavarian Government prior to permission. Permissions for motor boat usage (permit number 610-646-406), for water usage and field research station setup (permit number 610-641.6547) and for holding the test fish in on-site aquaculture facilities (permit number 4.13-568) were given by the rural district office Schwandorf. Permission for electrofishing was given by the rural district office Freising (permit number 31-1351-7562) and the fisheries rights owners (Fischereiverein e.V. Neunburg v. W., represented by president Michael Throner).

### Experimental design

Hatchery-reared fish were used in a standardized experiment, following the same procedure as described by Mueller, Pander & Geist (2017), to quantify hydropower-related internal injuries while accounting for the possibility of pre-damage occurring from aquaculture and transportation as well as naturally occurring anomalies. The experiment included seven native European fish species (Table 1). The seven fish species were chosen due to their morphology and their ecological importance in the study region (Mueller, Pander & Geist, 2017; Pander et al., 2018). Fish were directly delivered into rectangular fish tanks (3,000 mm × 700 mm × 700 mm, Aquacultur Fischtechnik GmbH, Nienburg, Germany) continuously supplied with water by a submersible pump (Easy-Mix-U20, Aquacultur Fischtechnik GmbH, Nienburg, Germany) at the study site, while being gradually adapted to the temperature and water chemistry. The experimental design is described in detail in Mueller, Pander & Geist (2017) and follows a group comparison approach to distinguish treatment effects from the control. In summary, “treatment fish” were released upstream of a hydropower facility with fish exclusion screen of 20 mm bar spacing and a Kaplan-turbine (for technical details of the hydropower facility see Knott et al. (2019)), and recaptured after turbine passage at 1 h emptying intervals with a knotless stow net of narrowing diameter and decreasing mesh size (length: 9.0 m, mesh sizes: 30 mm, 20 mm, 15 mm, 10 mm and 8 mm; for details see also Pander et al. (2018) and Knott et al. (2019)). “Sham fish” were released at the rectangular opening of the recovery net (Mueller, Pander & Geist, 2017; Knott et al., 2019). These fish were marked with a fin clip at the caudal fin to distinguish them from “treatment fish”, which were not marked. Wild fish moving downstream also caught in the net only occurred from the same species for European perch and roach and could be differentiated from experimental fish because of their specific size range as well as their smaller and less colorful pectoral and ventral fins. All fish were released gently in the water upstream of the power plant or recovery net using a water-filled 40 L bucket, by lowering the bucket to the water surface and lifting the bottom of the bucket using a rope. For a schematic of the release locations see Knott et al. (2019) and Mueller, Pander & Geist (2017). To account for pre-damage from breeding conditions, delivery to the study site and other handling effects (e.g., handling and catching from the fish tanks), but also naturally occurring anatomical anomalies, individuals from each species were investigated for injuries and mortality without any treatment (“control fish”, Mueller, Pander & Geist, 2017).

**Table 1:** Tested fish species.

Common name	Scientific name	Minimum total length	Maximum total length	Body shape	Swimmbladder morphology
European eel	*Anguilla anguilla* L.	220 mm	710 mm	Elongated	Physostome, one chamber
Barbel	*Barbus barbus* L.	40 mm	230 mm	Ventrally flattened	Physostome, two chambers
Roach	*Rutilus rutilus* L.	35 mm	220 mm	Laterally flattened	Physostome, two chambers
Danube salmon	*Hucho hucho* L.	90 mm	510 mm	Torpedo shaped	Physostome, one chamber
European grayling	*Thymallus thymallus* L.	65 mm	330 mm	Torpedo shaped	Physostome, one chamber
Brown trout	*Salmo trutta* L.	42 mm	410 mm	Torpedo shaped	Physostome, one chamber
European perch	*Perca fluviatilis* L.	54 mm	150 mm	Laterally flattened	Physoclist, one chamber

**Note:**

List of the tested fish species with their common names, scientific names, minimum and maximum total length, body shape and swim bladder type.

### External fish injury assessment

The assessment of externally visible fish injuries was carried out using the established protocol in Mueller, Pander & Geist (2017). Specifically, this assessment comprises 86 combinations of body parts with injury types as well as five general fish health criteria. Vitality, respiratory movements, parasitic infestations, fungal infections and nutritional status were checked first, followed by a systematic visual estimation of the different body parts for all other injury types. All investigators previously went through a whole day training on injury scoring and the use of the protocol. After evaluation, vital fish were transferred back to the fish tanks and kept in compartments (perforated plastic boxes of 60 × 40 × 44 cm; volume 70 L; Allibert Logic Box; Allibert Home, Villepinte, France) per treatment and species for 96 h to account for potential “delayed mortality”. Vital fish were released into the river downstream of the hydropower plant at the end of the experiment. All dead fish were frozen at −20 °C in zip-lock bags with an individual barcode and later evaluation of internal injuries (“immediate mortality” and “delayed mortality”). Severely damaged fish with minor vital functions (see Mueller, Pander & Geist, 2017) were euthanized using a tenfold overdose of MS 222 (Adam, Schürmann & Schwevers, 2013) and were also frozen. Additionally, a random reference sample of five fully vital fish from the groups “treatment fish”, “sham fish” and “control fish” was euthanized and frozen immediately after external injury evaluation (“immediate reference”) and after completion of the 96 h holding period (“delayed reference”).

### Internal fish injury assessment

**Figure 1:**
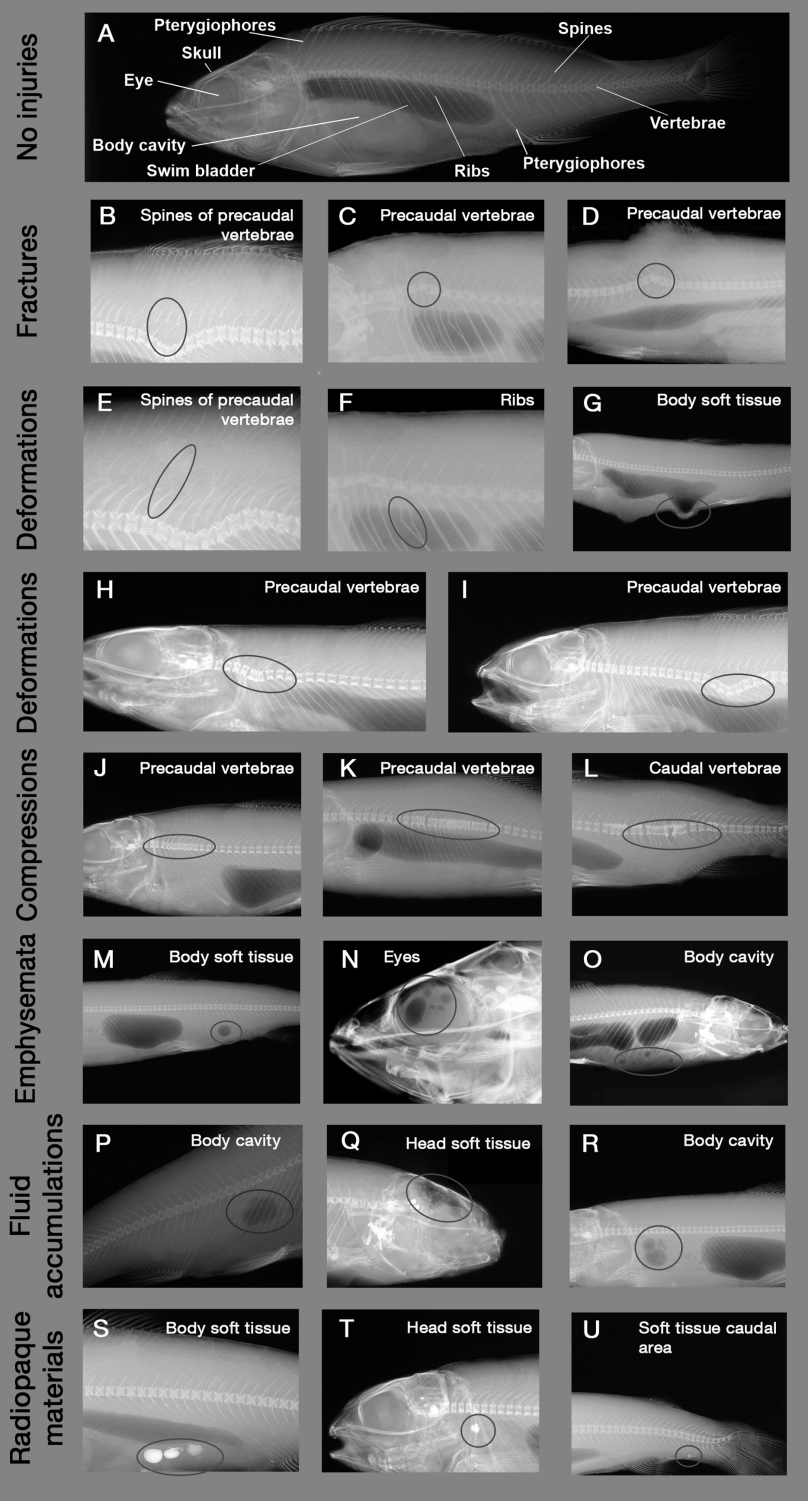
Example X-ray images of skeletal and soft tissue injuries. Encircled body parts indicate injured areas. (A) Reference fish with no injuries (*Perca fluviatilis*), (B–D) Fractures, (E–G) Deformations of spines, ribs and soft tissue, (H and I) Deformations of vertebrae, (J–L) Compressions, (M–O) Emboli, (P–R) Fluid accumulations, (S–U) Radiopaque materials. Source: Institute of Anatomy, Faculty of Medicine, LMU Munich, Munich, Bavaria, Germany.

**Figure 2:**
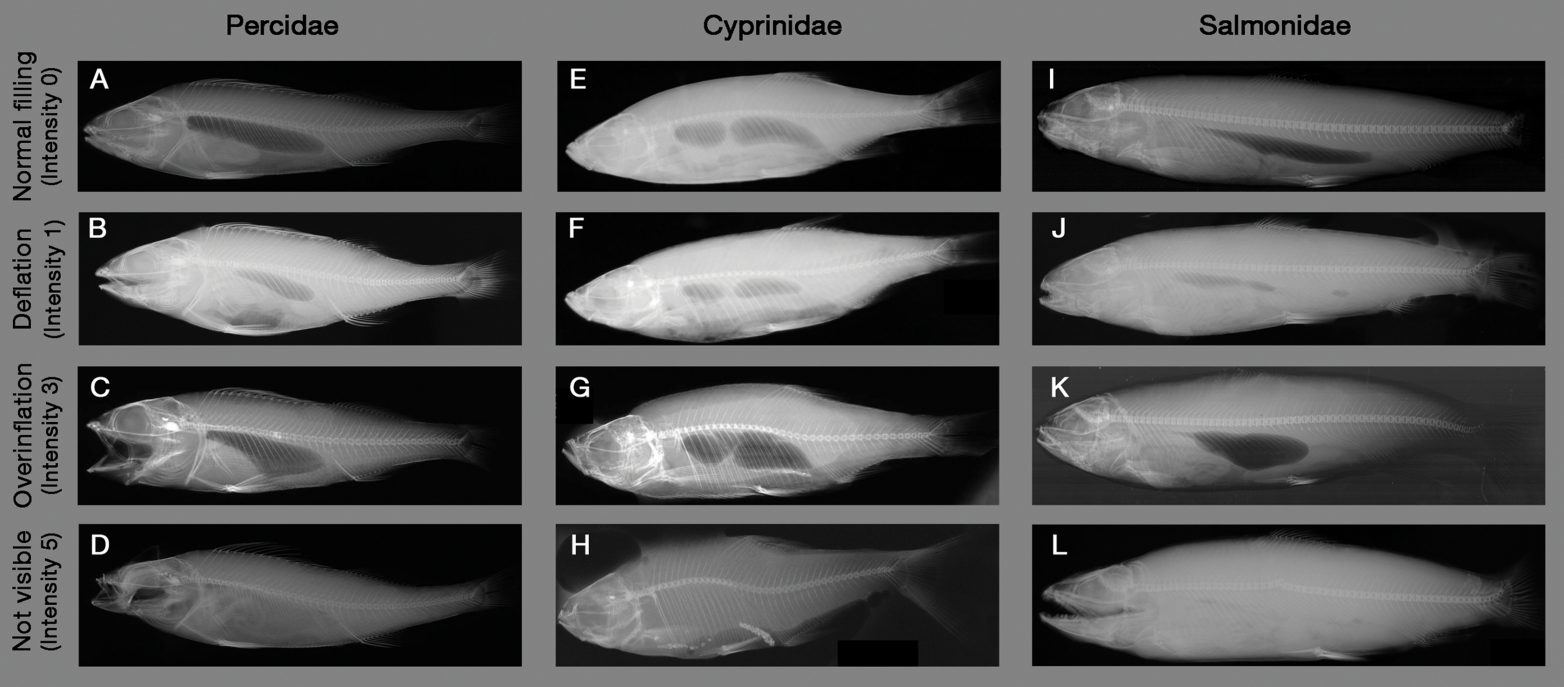
Example X-ray images of swim bladder anomalies in Cyprinids, Salmonids and Percids. (A, E and I) Normal swim bladder filling in Percidae, Cyprinidae and Salmonidae. (B–D) Different levels of swim bladder anomalies in Percidae, (F–H) different levels of swim bladder anomalies in Cyprinidae, (J–L) different levels of swim bladder anomalies in Cyprinidae. Source: Institute of Anatomy, Faculty of Medicine, LMU Munich, Munich, Bavaria, Germany.

Individual internal injuries of each frozen fish were observed by contact radiography (Figs. 1 and 2). The radiographies were conducted using an HP Cabinet X-Ray System (FAXITRON BIOPTICS, LLC, Tucson, AZ, USA) and DIN Agfa Structurix films (D4 DW ETE / D7 DW 18x24, Agfa-Gevaert N.V., Mortsel, Belgien, Europe) in the Anatomy Institute of the Ludwig-Maximilian University of Munich (LMU). A standard lateral view was taken of each fish as recommended by Wildgoose (2003). The peak voltage and duration of the radiographies depended on the individual morphology such as thickness and length (2.0 mA; 20 kV-20 s; 40 kV-20 s; 60 kV-60 s). X-ray films were developed and fixed for 7.4 min at 28 °C using a typon XS-2 developing machine and consecutively dried at 60 °C (developing bath: 10L H_2_0 at 15–40 °C, 5 L Structurix developer G135 A, 0.5 L Structurix G135 B, 0.5 L Structurix G135: fixation bath (Replenisher G335): 10 L H_2_O at 15–40 °C, 5 L Structurix G335 A and 1.25 L Structurix G335B mixed with 3.75 L H_2_O at 15–40 °C).

X-ray images were evaluated by a systematic visual screening using a dimmable A4 light table (M.way, Hongkong, China) and magnification glasses (6-fold and 12-fold magnification). The screening was started with an evaluation of the swim bladder to detect anomalies in filling state (rupture, overinflation, deflation or normal filling, see Fig. 2). Subsequently, the skeleton (skull, vertebrae, spines and ribs) was screened for fractures (i.e., contour disruptions) and deformations (i.e., shape changes) from the head to the caudal fin, followed by the pterygiophores. As a last step, a screening for fluid accumulations (more radiopaque), emphysema and free intraperitoneal gas (more radiolucent) was performed from the head to the caudal fin. For fluid accumulations, only sufficiently radiolucent fluid compared to surrounding tissue could be detected. X-ray images were evaluated by three persons, who were trained by experienced radiography experts (S. Milz, K. Sternecker), and underwent an intensive calibration meeting to assure comparability of results. Image evaluation was carried out as blinded experiment, with fish identified by their barcodes and evaluators not knowing to which experimental group they belong to.

Injury types are given in Table 2, with X-ray images in Fig. 1. Intensity of internal injuries was differentiated into four categories analogously as described for external injuries in Mueller, Pander & Geist (2017): no injury = 0, minor injury = 1, medium injury = 3 and severe injury = 5 (Table 3). For scoring the intensities of the injuries, distinct anatomical parts of fish body were investigated separately (skull, eyes, vertebrae, spines, ribs, pterygiophores, soft tissue, body cavity, swim bladder). Anatomical structures were named following Helfman et al. (2009) and Hastings, Walker & Galland (2015). The 36 possible combinations of anatomical structures with injury types were summarized in a protocol sheet, in which the intensity of each injury was recorded (Fig. A1). In the case of swim bladder anomalies, rupture (no swim bladder or contours of it visible) was defined as the most severe intensity = 5, overinflation was defined as intensity = 3, deflation as intensity = 1 and normal filling as intensity = 0 (Fig. 2). More details on the scoring of each injury type can be found in the score sheet in Table 3.

**Table 2:** Internal injury types.

Injury type	Description	Body parts
Fractures	Interruption of the continuity of skeleton elements and /or a loss of skeletal symmetry; disruption of outer skeletal contour	Skull, vertebrae, ribs, spines, pterygiophores
Deformations	Shift of vertebra resulting in a S-shape of the spine as well as unnatural turnaround or distortion of other bony structures	Skull, vertebrae, ribs, spines, pterygiophores
Compressions	Vertebra of the spine heavily squeezed together, also resulting in a deformation of ribs and spinous processes in the same area	Vertebrae
Emphysema	Blackish, often circular bubbles which are more radiolucent compared to fluid accumulations, with a clearly defined margin. Emphysema can appear in all soft parts of the fish body, often close to skeletal parts	Soft tissue
Free intraperitoneal gas	Blackish, often circular bubbles which are more radiolucent compared to fluid accumulations with a clearly defined margin, appearing in the body cavity	Body cavity
Fluid accumulations	Greyish areas which are more radiopaque compared to emphysema and free intraperitoneal gas, differing in shape and in contrast to emphysema and free intraperitoneal gas non-circular and with a less clearly defined margin	Soft tissue, body cavity
Swim bladder anomalies	Deflated or overinflated swim bladder with decreased size compared to natural conditions or no swim bladder visible anymore (rupture). Swim bladder anomalies often co-occur with free intraperitoneal gas due to tearing, rupture or gas exchange through the *ductus pneumaticus* in physostomes	Swim bladder
Radiopaque materials	Very bright areas on the X-ray film indicating material which is impermeable for radiation (e.g., pebbles taken up with the food)	Soft tissue, body cavity

**Note:**

List of the assessed injury types with description of their appearance and the body parts in which they were recorded.

**Table 3:** Scoring details for intensity evaluation of internal injuries.

	Fractures	Deformations	Compressions	Swim bladder anomalies	Emphysema/free intraperitoneal gas	Fluid accumulations	Radiopaque materials
Skull	0: no fractures	0: no deformation of bones					
	1: single bones partially fractured	1: single bones slightly deformed					
	3: several bones partially fractured or single bones totally fractured	3: several bones slightly deformed or single bones strongly deformed					
	5: major part of bones partially fractured, several bones totally fractured	5: major part of bones strongly deformed					
Eyes					0: no gas	0: no fluid accumulations	
					1: single small gas bubble of <5% of eye volume	1: fluid accumulations of <5% of eye volume	
					3: several small or single medium sized gas bubbles, volume >5% <20%	3: fluid accumulations of >5% <25% of eye volume	
					5: several medium sized or large gas bubbles, volume >20%	5: fluid accumulations of >25% eye volume	
Vertebrae	0: no fractures	0: no deformations	0: no compressions				
	1: one vertebra partially fractured but spine still in a continuous line	1: spine slightly bended from its natural shape	1: one vertebra compressed				
	3: up to three vertebrae partially fractured or disruption of outer skeletal contour	3: spine strongly bended from its natural shape	3: up to three vertebrae compressed				
	5: more than three partial fractures or total fracture of vertebral column, continuousity of spine clearly interrupted	5: spine heavily bended, for example, due to heavy fracture or double-bended in S-shape	5: more than three vertebrae compressed				
Spines,	0: no fractures	0: no deformation					
ribs and	1: one spine/rib/pterygiophore fractured	1: one spine/rib/pterygiophore deformed					
pterygio-phores	3: up to five spines/ribs/pterygio-phores fractured	3: up to five spines/ribs/pterygio-phores deformed					
	5: more than five spines/ribs/pterygio-phores fractured	5: more than five spines/ribs/pterygio-phores deformed					
Body cavity					0: no gas	0: no fluid accumulations	0: no radiopaque structures
					1: single small gas bubble of <5% of body cavity volume	1: fluid accumulations of <5% of head volume	1: single small radiopaque structures <3% of body cavity volume
					3: several small or single medium sized gas bubbles, volume >5% <20%	3: fluid accumulations of >5% <25% of body cavity volume	3: several small or single medium sized radiopaque structures, volume >3% <10%
					5: several medium sized or large gas bubbles, volume >20%	5: fluid accumulations of >25% body cavity volume	5: several medium sized or large radiopaque structures, volume >10%
Soft tissue					0: no gas	0: no fluid accumulations	0: no radiopaque structures
					1: single small gas bubble of <5% of body area volume	1: fluid accumulations of <5% of body area volume	1: single small radiopaque structures <3% of body area volume
					3: several small or single medium sized gas bubbles, volume >5% <20%	3: fluid accumulations of >5% <25% of body area volume	3: several small or single medium sized radiopaque structures, volume >3% <10%
					5: several medium sized or large gas bubbles, volume >20%	5: fluid accumulations of >25% body area volume	5: several medium sized or large radiopaque structures, volume >10%
Swim bladder				0: normal swim bladder filling			
				1: swim bladder strongly deflated but contours still visible, often accompanied by free intraperitoneal gas			
				3: swim bladder overinflated			
				5: no swim bladder and no contours visible, likely due to rupture			

### Statistical analyses

For each experimental group and injury type, the percentage of affected fish (injury incidence), the average injury intensity of all injured fish and of all fish were determined. Univariate statistics in the open-source software R (version 3.1.2, R Core Team, 2014) were used to compare injury intensities of all injured fish and all fish between experimental groups across all body parts and injury types.

To test for differences in internal fish injury patterns depictable with X-ray images between groups and species, a multivariate approach was used following the same general procedure as established in Mueller, Pander & Geist (2017) for external injuries. Specifically, for all multivariate analyses, a resemblance matrix using the Bray–Curtis Coefficient as a measure of similarity (Bray & Curtis, 1957) was generated from raw data on fish injury intensity. If one or more variables were entirely zero throughout all samples, a Bray–Curtis coefficient with zero-adjustment, was used including a dummy variable of the value 1 (Clarke, Somerfield & Chapman, 2006). Visualization was done by Metric MultiDimensional Scaling (MDS) of Bootstrap averages based on Bray–Curtis Similarities, grouping fish injury patterns for treatments and species.

Following the statistical injury evaluation procedure described in Mueller, Pander & Geist (2017) and the recommendations for complex experimental designs in Anderson, Gorley & Clarke, 2008, two-way and one-way PERMANOVAs (PERMutational ANalysis Of VAriance; Anderson, Gorley & Clarke, 2008) were run on Bray–Curtis Similarities to analyze the data for differences between internal injuries of different experimental groups and species. Similarity Percentages analysis (SIMPER; Clarke & Warwick, 2014) were used to test for differences in regularly occurring internal injury types which are contributing to between-group dissimilarities. Hierarchical clustering was used to analyze if there were jointly occurring external and internal fish injuries. The complete data set of external and internal injuries was used in the routine CLUSTER in PRIMER v7 (Clarke & Warwick, 2014) with group-average linking based on pairwise Bray–Curtis Similarities between variables (injuries). A dendrogram was generated to identify co-occurrence of the external and internal injuries. To test for significant groupings in the dendrogram, the SIMPROF routine was used (similarity profiles; Clarke & Warwick, 2014).

## Results

### General prevalence of internal fish injuries

Across all X-rayed fish, anomalies of the swim bladder (38% of all fish) as well as compressions (34%), deformations (30%) and fractures (28%) of vertebrae were the most commonly observed internal injury types, succeeded by deformations of ribs and spines (25%), free intraperitoneal gas (20%), deformations of pterygiophores (15%), fractures of ribs and spines (14%) and fractures of pterygiophores (10%, Table 2). Fluid accumulations and emphysema in the connective tissue and internal injuries of the head occurred less frequently (Table 2). Fluid accumulations in the eyes as well as radiopaque material or fluid accumulations in the soft tissue of the head were the rarest injury types or observations, only affecting 1% or less of all fish (Table 2).

### Determination of turbine effects

**Figure 3:**
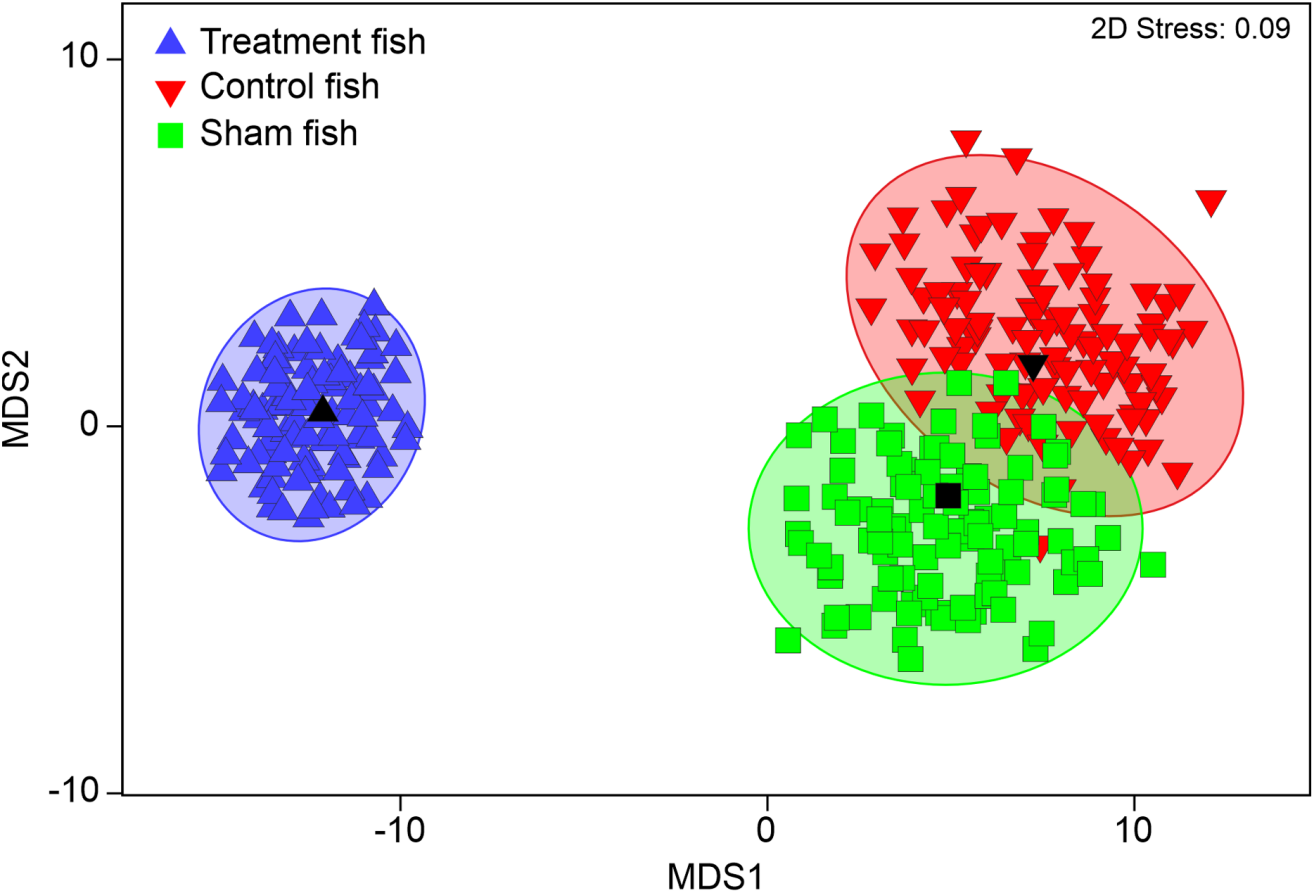
Multivariate internal injury patterns in different treatments. Metric multidimensional scaling (MDS) of bootstrap averages per experimental group (“control fish”, “sham fish”, “treatment fish”) from internal fish injuries in all seven hatchery-reared fish species tested in the standardized experiment. Bootstrap averages are based on pairwise Bray–Curtis Similarities between each pair of fish calculated from internal injury intensities. “Control fish”: hatchery-reared fish without further treatment, *n* = 137, “Sham fish”: hatchery-reared fish directly released at the entrance of the stow net, *n* = 171, “Treatment fish”: hatchery-reared fish released upstream of the fish protection screen, making them pass the entire power plant including screen and turbine, *n* = 594.

PERMANOVA detected significant differences in internal injury patterns of “treatment fish” after power plant passage compared to “sham fish” and “control fish” (pseudo-*F* = 14.1, d.f. = 2, *P* < 0.001; “treatment fish”–“sham fish”: *t* = 4.0, d.f. = 717, *P* < 0.001; “treatment fish”–“control fish”: *t* = 4.0, d.f. = 717, *P* < 0.001), but no specific patterns of internal injuries could be related to fish handling (“sham fish”–“control fish” *t* = 0.9, d.f. = 294, *P* = 0.59; Fig. 3). For instance, incidence of vertebrae fractures was increased four-fold, incidence of fluid accumulations in the soft tissue and body cavity was double and incidence of swim bladder anomalies was also almost double after turbine passage (Table 4).

**Table 4:** Internal injury intensity and incidence in different experimental groups.

	Control fish					Sham fish					Treatment fish				
	Int all	±SD	Int in.	±SD	Inc.	Int all	±SD	Int in.	±SD	Inc.	Int all	±SD	Int in.	±SD	Inc.
Swim bladder anomalies	0.6	±1.3	2.2	± 1.7	27.7	0.7	±1.6	2.7	± 1.9	26.9	1.4	±2.0	3.3	±1.9	43.6
Vertebrae fractures	0.0	±0.1	0.3	± 0.0	5.1	0.1	±0.3	0.7	± 0.8	9.4	0.5	±0.9	1.4	±1.0	38.4
Vertebrae compressions	0.2	±0.5	0.8	± 0.6	23.4	0.2	±0.4	0.7	± 0.5	28.1	0.3	±0.5	0.7	±0.5	38.4
Vertebrae deformations	0.1	±0.2	0.5	± 0.3	19.7	0.1	±0.3	0.7	± 0.5	17.5	0.3	±0.5	0.7	±0.7	35.5
Ribs/spines deformations	0.1	±0.2	0.5	± 0.3	17.5	0.1	±0.2	0.5	± 0.3	20.5	0.2	±0.4	0.7	±0.5	27.4
Free intraperitoneal gas	0.3	±0.9	2.1	± 1.2	16.1	0.2	±0.7	1.7	± 1.1	14.0	0.5	±1.1	2.1	±1.2	22.1
Ribs/spines fractures	0.0	±0.1	0.4	± 0.1	3.6	0.0	±0.1	0.3	± 0.0	5.3	0.2	±0.6	1.3	±0.7	18.5
Pterygiophores deformations	0.2	±0.5	1.2	± 0.6	16.8	0.2	±0.4	1.1	± 0.4	18.7	0.2	±0.5	1.2	±0.8	13.6
Pterygiophores fractures	0.1	±0.2	1.0	±0	6.6	0.1	±0.2	1.0	±0.0	5.3	0.4	±1.3	3.3	±1.9	11.8
Body cavity fluid accumulations	0.1	±0.4	1.5	±0.9	5.8	0.0	±0.3	1.3	±0.8	3.5	0.2	±0.7	2.1	±1.4	8.9
Body soft tissue fluid accumulations	0.0	±0.2	0.8	±0.4	3.6	0.0	±0.1	0.6	±0.2	4.1	0.1	±0.3	0.8	±0.6	8.1
Body cavity radiopaque materials	0.2	±0.6	1.5	±0.9	13.9	0.3	±1.0	2.6	±1.6	11.1	0.1	±0.5	1.5	±1.1	7.2
Skull fractures	0.0	±0.1	1.0	NA	0.7	0.0	±0.4	2.3	±2.3	1.8	0.1	±0.7	2.6	±1.4	5.6
Body soft tissue emphysema	0.0	±0.1	1.0	±0.7	1.5	0.0	±0.2	1.0	±0.8	3.5	0.0	±0.2	0.7	±0.4	4.7
Head soft tissue emphysema	0.1	±0.3	1.3	±0.8	5.1	0.1	±0.4	1.5	±0.9	4.7	0.0	±0.3	1.4	±0.8	3.5
Body soft tissue radiopaque materials	0.0	±0.2	0.8	±0.5	5.1	0.0	±0.1	0.5	±0.0	5.8	0.0	±0.1	0.6	±0.2	2.2
Skull deformation	0.0	±0.1	1.0	NA	0.7	0.0	±0.4	2.0	±2.0	2.3	0.1	±0.4	2.8	±1.7	1.9
Eyes emphysema	0.0	±0.0	NA	NA	0.0	0.0	±0.0	NA	NA	0.0	0.1	±0.5	3.2	±1.4	1.9
Head soft tissue fluid accumulations	0.0	±0.0	NA	NA	0.0	0.0	±0.1	1.0	±0.0	1.2	0.0	±0.1	1.0	±0.0	1.2
Head soft tissue radiopaque materials	0.0	±0.0	NA	NA	0.0	0.0	±0.1	1.0	±0.0	1.2	0.0	±0.1	1.0	±0.0	0.7
Eyes fluid accumulations	0.0	±0.1	1.0	NA	0.7	0.0	±0.0	NA	NA	0.0	0.0	±0.1	1.0	±0.0	0.3

**Note:**

Table summarizing injury intensity and incidence for the experimental groups “Control fish” = fish after delivery from hatchery (*n* = 137), “Sham fish” = fish released directly at the entrance of the stow net (*n* = 171), “Treatment fish” = fish released upstream from fish protection screen (*n* = 594). Int_all_ = arithmetic mean of injury intensity across all individuals, Int_in_ = arithmetic mean of injury intensity across injured individuals, Inc. = incidence in %/percentage of affected individuals for each of the treatments, SD, standard deviation. The shading of the table in different intensities of red indicates increasing injury intensity/incidence with increasing intensity of red. The shading is graded separately for intensity and incidence due to the different numerical scale of both measures.

**Figure 4:**
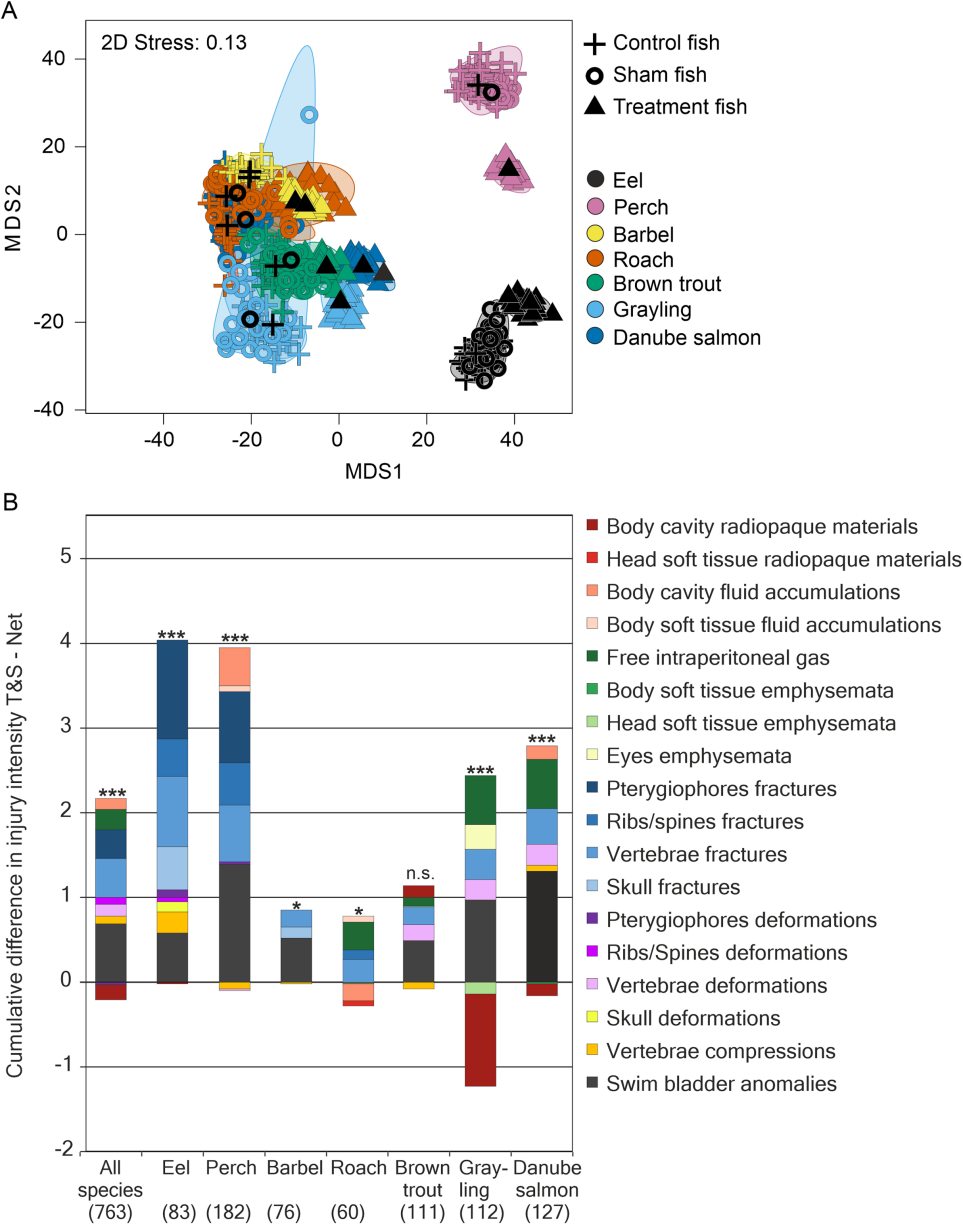
Species–specific differences in internal fish injury patterns between treatments. (A) Metric multidimensional scaling (MDS) of bootstrap averages per experimental group and fish species from internal injuries. Bootstrap averages are based on pairwise Bray–Curtis Similarities between each pair of fish calculated from internal injury intensities. Different symbols indicate different experimental groups, different colors indicate different species (Eel: *n* = 107, Perch: *n* = 205, Barbel: *n* = 93, Roach: *n* = 80, Brown trout: *n* = 136, Grayling: *n* = 135, Danube salmon: *n* = 146). “Control fish”: hatchery-reared fish without further treatment, “Sham fish”: hatchery-reared fish directly released at the entrance of the stow net, “Treatment fish”: hatchery-reared fish released upstream of the fish protection screen, making them pass the entire power plant including screen and turbine. (B) Absolute differences in internal injury intensity between experimental groups “Sham fish” and “Treatment fish” (plotted cumulatively) for internal injuries with a contribution to between-group dissimilarity larger than 5% according to similarity percentage analysis (SIMPER). The size of the bar parts indicates the delta in intensity values for the respective injury type. Cum.%, cumulative contribution to between-group dissimilarity according to SIMPER. Contribution to between-group dissimilarity of single injury types was ranging between 3% and 67%, Diss/SD ranging between 0.11 and 1.40. Numbers in brackets below the species labels of the barplots indicate degrees of freedom in PERMANOVA comparisons, asterisks above the barplots indicate significant differences between the compared treatments “Sham fish” and “Treatment fish”, with significance levels coded as follows: *P < 0.05, **P < 0.01, ***P < 0.001.

Internal injuries were also species-specific (“control fish”–“sham fish”: pseudo-*F* = 38.7, d.f. = 6, *P* < 0.001) and a significant interaction term between the factors “group” and “species” indicated differences in the severity of turbine passage effects among species (pseudo-*F* = 2.1, d.f. = 12, *P* < 0.001, Fig. 4). While fractures of skeletal parts were most pronounced in eel, fluid accumulations and swim bladder anomalies strongly contributed to turbine effects in perch and free intraperitoneal gas were increased after turbine passage in grayling and Danube salmon (Fig. 3). The most pronounced differences in internal injuries between “control fish” and “sham fish” were detected for European eel (*t* = 2.3, d.f. = 83, *P* < 0.001), European perch (*t* = 2.5, d.f. = 182, *P* < 0.001) and European grayling (*t* = 2.6, d.f. = 112, *P* < 0.001), followed by Danube salmon (*t* = 2.8, d.f. = 127, *P* < 0.001; Fig. 4). Number of injuries, incidence and injury intensity were higher in “treatment fish” than in “sham fish” and “control fish” (Table 4).

### Internal injuries, mortality and delayed effects

**Figure 5:**
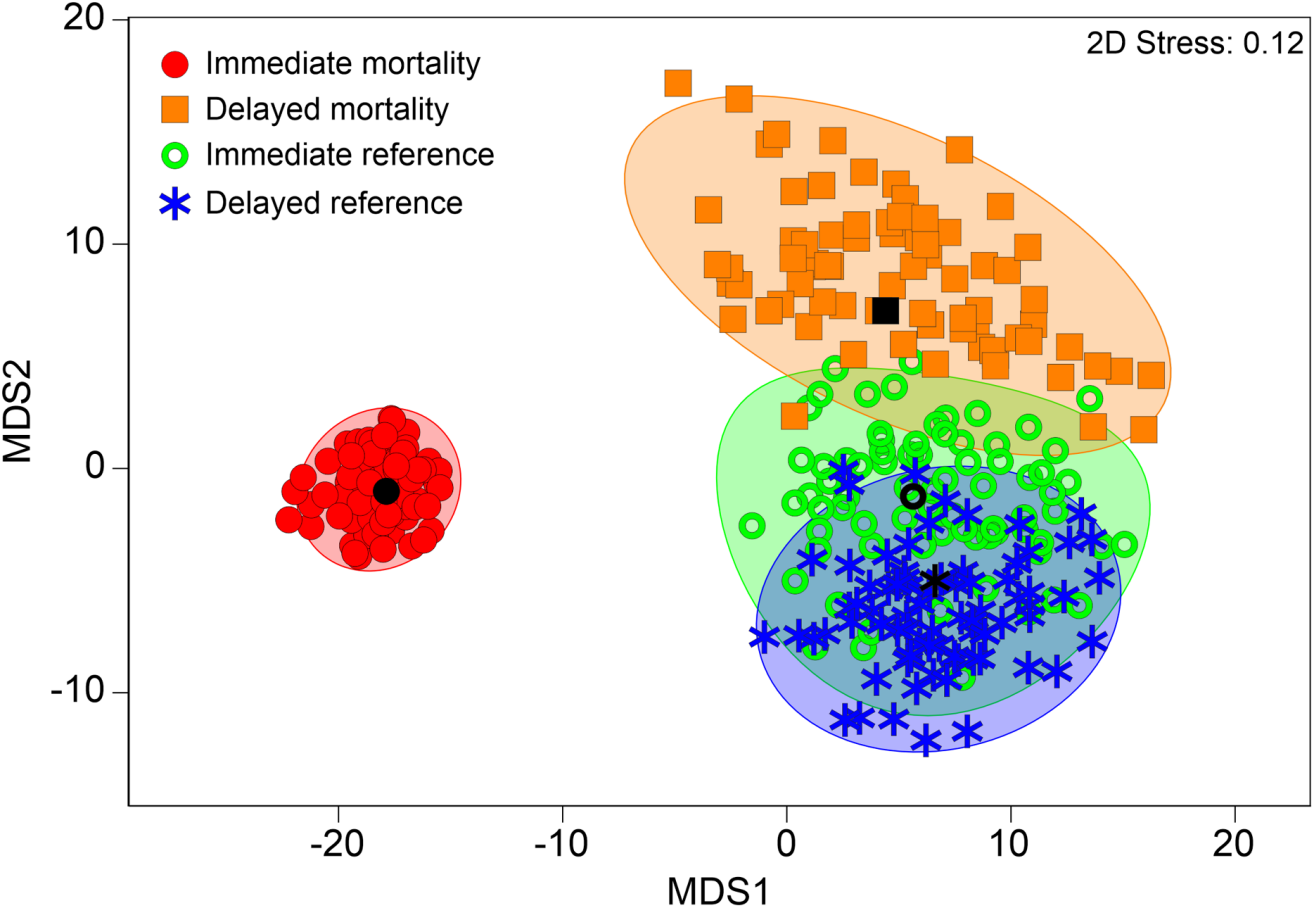
Multivariate internal fish injury patterns in references and mortalities. Metric multidimensional scaling (MDS) of bootstrap averages per group (“immediate mortality”, “delayed mortality”, “immediate reference”, “delayed reference”) from internal fish injuries in all seven hatchery-reared fish species tested in the standardized experiment. Bootstrap averages are based on pairwise Bray–Curtis Similarities between each pair of fish calculated from internal injury intensities. “immediate mortality”: fish that were immediately dead after power plant passage, *n* = 417; “delayed mortality” = fish that immediately survived power plant passage but died during the 96 h maintainance period, *n* = 40; “delayed reference” = fish that survived power plant passage and the 96 h maintainance period, euthanized using 10-fold overdose of MS 222, *n* = 64; “immediate reference” = fish that survived power plant passage and were immediately euthanized as a reference sample, *n* = 73.

**Figure 6:**
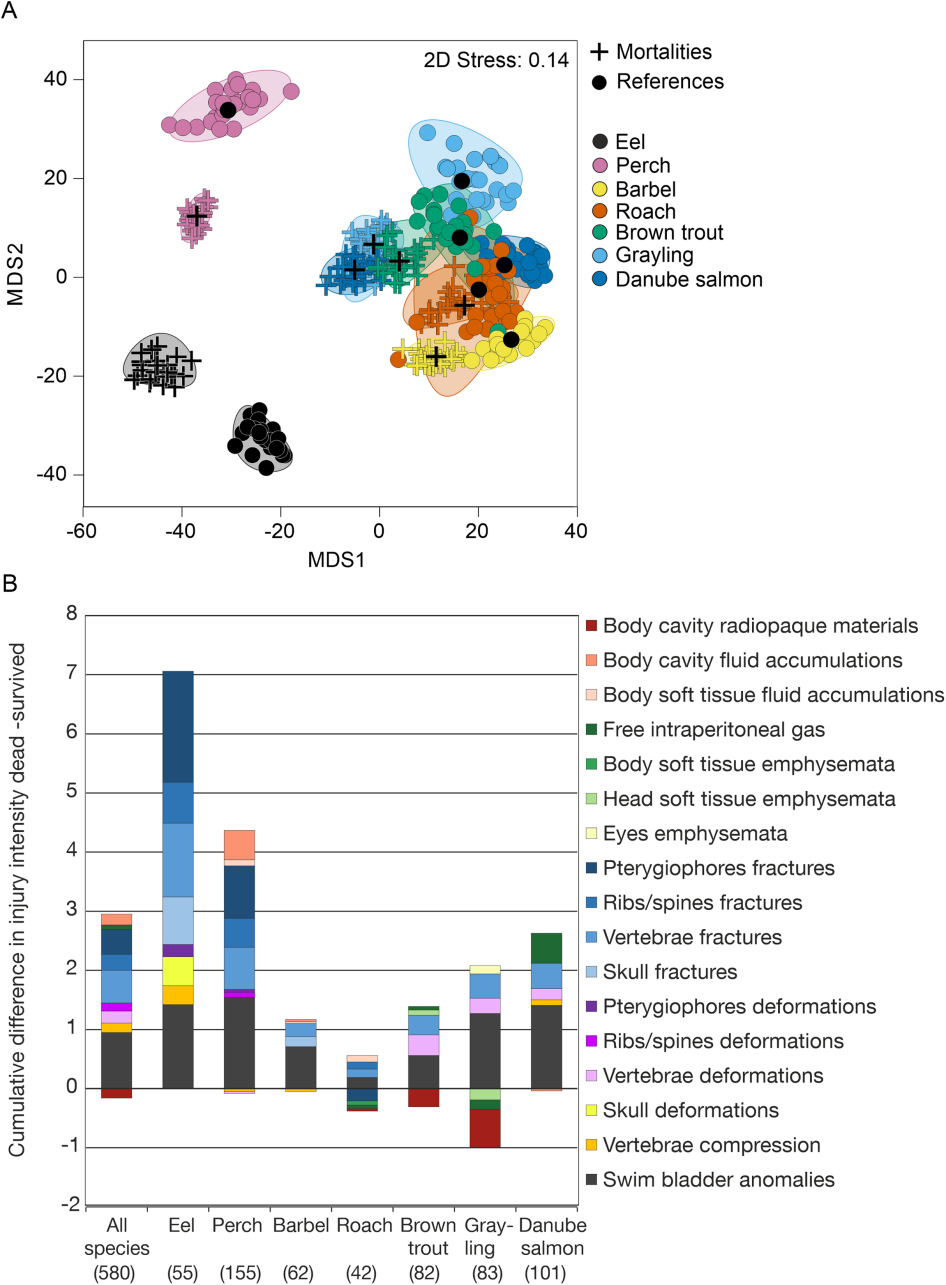
Species-specific differences in internal injury patterns between references and mortalities. (A) Metric multidimensional scaling (MDS) of bootstrap averages per group (“immediate mortality”, “delayed mortality”, “immediate reference”, “delayed reference”) from internal fish injuries in each fish species from “Treatment fish”. Bootstrap averages are based on pairwise Bray–Curtis Similarities between each pair of fish calculated from internal injury intensities. Different symbols indicate different groups, different colors indicate different species (Eel: *n* = 57, Perch: *n* = 157, Barbel: *n* = 64, Roach: *n* = 44, Brown trout: *n* = 84, Grayling: *n* = 85, Danube salmon: *n* = 103). “Immediate mortality”: fish that were immediately dead after power plant passage; “delayed mortality” = fish that immediately survived power plant passage but died during the 96 h maintainance period, “delayed reference” = fish that survived power plant passage and the 96 h maintainance period, euthanized using 10-fold overdose of MS 222, “immediate reference” = fish that survived power plant passage and were immediately euthanized as a reference sample. (B) Absolute differences in internal injury intensity between mortalities (“immediate mortality” and “delayed mortality”) and references (“immediate reference” and “delayed reference”) (plotted cumulatively) for internal injuries with a contribution to between-group dissimilarity larger than 5% according to similarity percentage analysis (SIMPER). The size of the bar parts indicates the delta in intensity values for the respective injury type. Cum.%, cumulative contribution to between-group dissimilarity according to SIMPER. Contribution to between-group dissimilarity of single injury types was ranging between 3% and 59%, Diss/SD ranging between 0.21 and 1.11. Numbers in brackets below the species labels of the barplots indicate degrees of freedom in PERMANOVA comparisons, asterisks above the barplots indicate significant differences between dead fish and survivors, with significance levels coded as follows: *P < 0.05, **P < 0.01, ***P < 0.001.

Internal injury patterns of fish with “immediate mortality” after turbine passage strongly differed from those of fish that survived hydropower plant passage (“immediate reference”) according to PERMANOVA (pseudo-*F* = 7.54, d.f. = 3, *P* < 0.001; “immediate mortality”–“immediate reference”: *t* = 3.1, d.f. = 476, *P* < 0.001; “immediate mortality”–“delayed reference”: *t* = 3.6, d.f. = 468, *P* < 0.001, Fig. 5). MDS of bootstrap averages also indicates lowest variability of internal injury patterns in fish killed by the power plant (Fig. 5). Injury patterns of fish with “delayed mortality” within 96 h after turbine passage significantly differed from those of fish with “immediate mortality” (PERMANOVA: “delayed mortality”–“immediate mortality”: *t* = 1.8, d.f. = 444, *P* < 0.0, Fig. 5). The number of internal injuries was almost double in dead fish compared to survived fish (dead: 4.1 ± 3.5, survived: 2.1 ± 2.2, Wilcox-test: *W* = 38538, *P* ≤ 0.01) and also higher in fish with “immediate mortality” (4.3 ± 3.5) compared to fish with “delayed mortality” (2.9 ± 3.0). Similar results were found for injury intensity, which was three-fold in fish with “immediate mortality” or “delayed mortality” compared to the “immediate reference” or “delayed reference” (mortality: 9.9 ± 10.6, reference: 3.4 ± 4.3, Wilcox-test: *W* = 39750, *P* ≤ 0.01). According to SIMPER analysis, injuries discriminating between mortality and reference were highly similar to those discriminating between “sham fish” and “treatment fish”, with fractures of skeletal parts and swim bladder anomalies being characteristic for mortalities in all species (Fig. 6). Species–specific contributions in SIMPER were also similar to those found for group comparisons, with fractures being particularly relevant in eel, fluid accumulations in perch and free intraperitoneal gas in salmonids (Fig. 6). In addition to internal injuries that were more intense in dead fish (“immediate mortality” and “delayed mortality”), fish that survived turbine passage (“immediate reference” and “delayed reference”) more frequently had radiopaque material in their body cavity. This was particularly pronounced in European grayling and brown trout (Fig. 6). Fish with “delayed mortality” for instance had increased intensity of free intraperitoneal gas, compressions of vertebrae, deformations of skeletal elements and fluid accumulations compared to the “delayed reference”, but less radiopaque material in the body cavity (Contribution >5% according to SIMPER). Compared to fish with “immediate mortality” after power plant passage, those with “delayed mortality” particularly had a less average intensity of anomalies of the swim bladder and of vertebral fractures and deformations (Contribution >5% according to SIMPER).

### Links between externally visible and internal injuries

**Figure 7:**
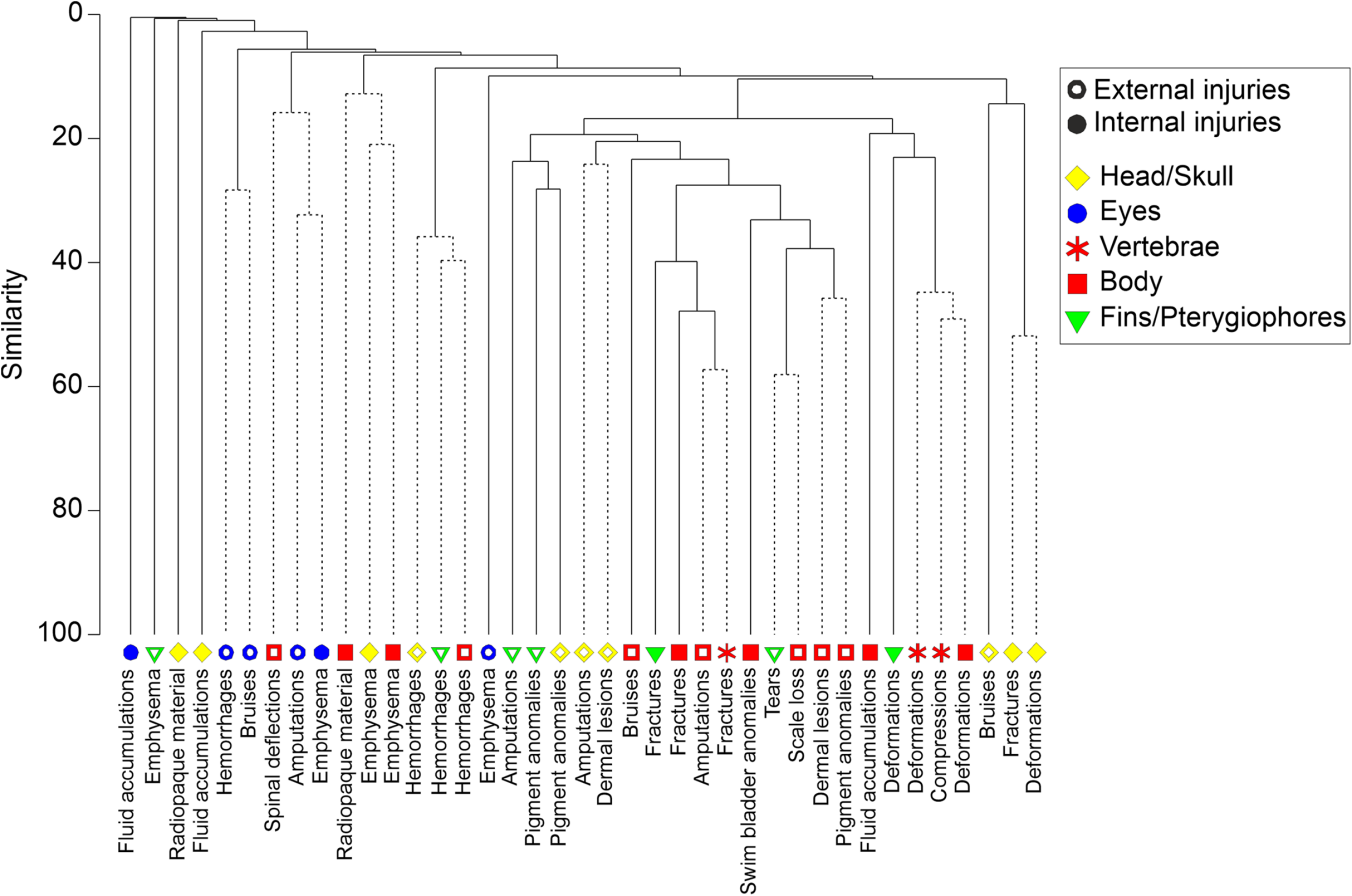
Dendrogram of internal and external fish injury co-occurance. Dendrogram for hierarchical clustering (using group-average linking) of internal and external injury types by body part, based on Bray–Curtis Similarities (cophenetic correlation = 0.86, *P* ≤ 0.05). Dashed lines indicate that there is no further significant substructure according to SIMPROF test. Open symbols indicate external injuries, filled symbols indicate external injuries. Different body parts are distinguished by symbols and colors.

Cluster analysis generally revealed low co-occurrence of externally visible and internal injuries (Fig. 7, cophenetic correlation = 0.87, *P* < 0.05). Bruises and amputations of body parts were the only externally visible injuries that coincided with internal injuries at more than 20% similarity (fractures of vertebrae, body skeleton and pterygiophores) or within a cluster without significant substructure according to SIMPROF test (bruises at the head and fractures or deformations of the skull, Fig. 7). However, still 29% of all fish with fractures of vertebrae had no externally visible indication of such severe injury (e.g., amputations or bruises). The internal injury types fractures, deformations and compressions generally occurred jointly in different body parts (Fig. 7). Specifically in perch, swim bladder deflations or ruptures frequently coincided with vertebral injuries, where 80% of all individuals with swim bladder injuries were also observed to have fractures of vertebrae.

## Discussion

Previous applications of X-ray imaging where mostly highly specific to a small number of species and injuries (e.g., barotrauma in sturgeon: Brown et al., 2013; barotrauma, emphysema and hemorrhages in three species: Brown, Walker & Stephenson, 2016). The novelty of the X-ray based assessment of turbine passage effects on internal fish injuries presented herein is that it covers swim bladder anomalies as well as skeletal and soft tissue injuries in seven species and considers a systematic visual screening. A systematic visual screening of X-ray images can help to determine different injury types related to turbine passage and achieve a higher comparability between studies on hydropower effects. This facilitates comparing effects on different species as well as comparisons among different hydropower and operation types.

### Species specific differences and determination of turbine effects

The internal injuries detected using the proposed X-ray-based assessment protocol could clearly be assigned to specific physical processes in the turbine, such as swim bladder rupture and emphysema which can be caused by sudden changes of pressure (Boys et al., 2016; Pflugrath, Boys & Cathers, 2018), or fractures of vertebrae and other injuries of the skeleton which can be caused by blade strike (Saylor, Fortner & Bevelhimer, 2019), severe turbulences or fluid shear (Neitzel et al., 2004; Deng et al., 2005). The species-specific susceptibility to different injury types, similarly to external injuries (Mueller, Pander & Geist, 2017; Pander et al., 2018; Bierschenk et al., 2019; Knott et al., 2019), can be explained by species morphology and internal anatomy and also reiterate what others have highlighted previously, that it is difficult to use one species as a proxy of the potential for impacts (Brown et al., 2014; Beirão et al., 2018; Silva et al., 2018). For instance, European eel and European perch in this study were both heavily affected by turbine passage, but the types of injuries were different. Due to its size and elongated body shape European eel were most prone to blade-strike (Ferguson et al., 2008) and the resulting fractures of vertebrae, while the tested European perch had a lower collision risk due to their relatively small maximum body size used (150 mm). However, European perch is a physoclist species without a connection (*Ductus pneumaticus*) between swim bladder and esophagus, making it very prone to swim bladder rupture if exposed to very rapid pressure changes (Abernethy, Amidan & Čada, 2001). During turbine passage, fish are exposed to rapid decompression (Colotelo et al., 2012), which causes an increase in swim bladder volume following Boyle’s Law. Therefore, gas exchange in the swim bladder of physoclists is relatively slow and a rapid increase in volume is more likely to result in rupture than physostomous species such as salmonids or cyprinids (Blaxter & Batty, 1990, Boys et al., 2016; Beirão et al., 2018; Pflugrath, Boys & Cathers, 2018; Pflugrath et al., 2019). American eel have been found to be exceptionally effective at expelling gas from the swim bladder which resulted in a very low susceptibility to barotrauma (Pflugrath et al., 2019) and this is also likely the case for European eel (Turnpenny et al., 1992). This may also explain our observation that many of the investigated eel from all experimental groups, also including “sham fish” and “control fish”, showed empty swim bladders on the X-ray images. However, results from grayling and Danube salmon with increased intensities and frequencies of swim bladder anomalies and free intraperitoneal gas indicate that pressure changes may also affect physostomous species.

A surprising finding was that radiopaque material in salmonids was regularly more present in fish that survived turbine passage than in those that died. In this case, radiopaque material were pebbles that were probably taken up by the fish with the food. So far it seems unclear how the presence of pebbles can be related to survival in the turbine, but there may be indirect effects (e.g., related to gravitation, buoyancy or decompression) helping the fish to better survive. In terms of barotrauma it would be expected that these fish would be more dense due to the weight of the pebbles and require more gas in the swim bladder to maintain buoyancy. This added gas in the swim bladder should make them more prone to rupture. However, it needs to be considered here that the majority of fish in this study are not likely depth acclimated since they were directly introduced in the turbine intake from surface acclimation in the fish tanks. Therefore, the presence of pebbles in the gastrointestinal tract may have different results if the fish were depth acclimated prior to passage through the turbine. This effect needs further validation, but if this is repeatedly observed, further investigation is warranted.

### Internal injuries, mortality and delayed effects

Direct mortality was clearly related to severe injuries such as vertebral fractures, which likely resulted from blade-strike, collision with other structures of the turbine or exposure to fluid shear, or swim bladder rupture which likely resulted from rapid pressure changes. Delayed mortality was related to less severe injuries such as skeletal deformations, emphysema, free intraperitoneal gas or fluid accumulations, while fractures of bony structures and swim bladder rupture were less frequent. It is logical that fish do not immediately die from these injuries, but they can suffer from them in the long-term and often did not survive the 96 h holding period. Under natural conditions, these injuries may, for example, lead to movement disorders or internal inflammations which can potentially make them more prone to predation (Brown et al., 2014). The high occurrence rate of free intraperitoneal gas of salmonids with delayed mortality indicates that these species may be more susceptible to barotrauma issues than would be expected for a physostomous species with a single chamber swim bladder and their capacity for rapid gas evacuation from the swim bladder. There is potential that, if gas emboli or emphysema were observed in locations other than within the body cavity, these are being caused by the physics described by Henry’s Law, where gas dissolved in the blood or other bodily fluids comes out of solution.

### Links between externally visible and internal injuries

Internal and external injuries had a higher association in individuals with very clear signs of severe blade strike, showing amputations of body parts externally and fractures of vertebrae on the X-ray image, but there were only weak relations among other injury types. Remarkably, one third of all individuals with fractures of vertebrae had no externally visible signs of severe injury, which can likely be attributed to exposure to fluid shear (Pflugrath et al., 2020). Conversely, occasional deformations of the vertebral column were indicated during the external evaluation but could not be confirmed through the X-ray image, suggesting the occurrence of false positives. These observations clearly indicate that even an assessment of severe injuries should not be limited to external evaluation. Furthermore, certain injuries may result in others which can be resolved by studying the coincidence of injuries. For instance, this was evident for swim bladder rupture, particularly in physoclist perch, where, when exposed to rapid decompression, the swim bladder rapidly expands within the fish’s body and can cause fractures of vertebrae or other skeletal injuries (Rummer & Bennett, 2005).

A combination of external injury evaluation, internal injury evaluation and delayed effect assessment seems particularly relevant for the monitoring of turbine and pump passage effects, where internal injuries are highly likely to occur. Obviously, neither internal injuries of any severity nor changes of survival can be predicted from an external inspection directly after turbine passage.

## Conclusions and Recommendations

The X-ray screening method for internal fish injuries applied herein may also be useful for laboratory experiments such as barotrauma and fluid shear studies which are currently carried out on different species worldwide to better predict the effects of pressure changes on fish in relation to passage of different turbine types. The X-ray method can be very valuable in this context, since a fish can be examined internally without the need to be euthanized, if a portable X-ray machine is available (see Bowersox et al., 2016). This would allow for an extended holding period post exposure and assessment of delayed effects, without the need to decide for either an assessment of immediate or delayed effects. A further advantage of being minimally invasive is that the X-ray method has a minimum risk for preparation artifacts compared to necropsy. However, since there are injuries that can be seen in a necropsy (e.g., injuries of the heart, spleen) that can’t be seen in the X-ray and vise versa (particularly injuries of the skeleton) it may be valuable to combine the X-ray method with a final necropsy at the end of the holding period.

The minimal invasive nature of the X-ray method is also exceptionally important for examining wild fish in the field, which may not only suffer from hydropower effects but also from other stressors that can cause internal injuries. This can for instance be diseases (e.g., swim bladder rupture due to infections or tumors: Wildgoose, 2003, skeletal damage due to whirling disease: Hoffman, Dunbar & Bradford, 1962), genetic defects (e.g., skeletal deformations: Gorman, Tredwell & Breden, 2007; Porta & Snow, 2019), chronic exposure to insecticides (e.g., anomalies of the vertebral column due to toxaphene exposure: Smith & Smith, 1994), attempted predation effects or regular handling in the context of fisheries management (e.g., electrofishing-related spinal injuries: Sharber & Carothers, 1988; Hollender & Carline, 1994). The X-ray evaluation procedure used herein covers an evaluation of the effects of all those stressors in a standardized way and could therefore ideally serve for an assessment of fish health and welfare in wild populations.

It would be helpful for future studies to generally have a larger body of standardized information on natural X-ray morphology of different fish species. This knowledge is currently very scarce in fish compared to humans or pet animals and could easily be generated by using the protocol presented herein, possibly complemented by some additional standardized measurements (e.g., swim bladder size, angle and distance of specific bones) and by automation of image evaluation, for example, applying machine learning. For a balanced view on pros and cons, it has to be noted that the internal injury assessment applied herein provides a high throughput method, but there may be injuries missed due to limitations of the X-ray method (lack of differences in density in soft tissue and organs, 2D vision with exclusive consideration of lateral view and visual evaluation of X-ray images). Other diagnostic imaging methods such as CT or MRI should also be considered if injuries in specific organs are of interest (Rogers et al., 2008; Roach, Hall & Broadhurst, 2011).

## Supplemental Information

10.7717/peerj.9977/supp-1 Supplemental Information 1 Example of internal injury protocol sheet.

10.7717/peerj.9977/supp-2 Supplemental Information 2 Raw data file on internal and external fish injuries.

## Additional Information and Declarations

### Field Study Permissions

The following information was supplied relating to field study approvals (i.e., approving body and any reference numbers): Permissions for motor boat usage (permit number 610-646-406), for water usage and field research station setup (permit number 610-641.6547) and for holding the test fish in on-site aquaculture facilities (permit number 4.13-568) were given by the rural district office Schwandorf. Permission for electrofishing was given by the rural district office Freising (permit number 31-1351-7562) and the fisheries rights owners (Fischereiverein e.V. Neunburg v. W., represented by president Michael Throner).

### Data Availability

The following information was supplied regarding data availability: The raw data of all external and internal injuries of every single fish are available as a Supplemental File.
