# Supplementary material for: Assessing turbine passage effects on internal fish injury and delayed mortality using X-ray imaging
Source: PeerJ. 2020 Sep 16;8:e9977. doi: 10.7717/peerj.9977 (PMC7501806; doi:10.7717/peerj.9977)
Supplement: Supplemental Information 1 [file peerj-08-9977-s001.pdf]

A rapid x-ray screening method to determine internal fish injury and causes of delayed mortality after hydropower turbine passage (animal care permit number: AZ 55.2-1-55-2532-31-15)

Lehrstuhl für Aquatische Systembiologie

Internal fish injury protocol

Site: ________________ **Date of x-ray:** _______________ **Settings:** _____ kV, _____ sec

Species: ☐ Brown trout ☐ Grayling ☐ Danube salmon ☐ Eel ☐ Barbel ☐ Perch ☐ Roach **Fish ID**: _______________

	Fractures	Deformations	Compressions	Swim bladder anomalies	Emphysema/ free intraperi-toneal gas	Fluid accumulations	Radiopaque materials
Skull			X	X	X	X	X
Head soft tissue	X	X	X	X			
Eyes	X	X	X	X			X
Precaudal vertebrae				X	X	X	X
Spines of precaudal vertebrae			X	X	X	X	X
Ribs			X	X	X	X	X
Pterygiophores			X	X	X	X	X
Body cavity	X	X	X	X			
Body soft tissue	X	X	X	X			
Swim bladder	X	X	X		X	X	X
Caudal vertebrae				X	X	X	X
Spines of caudal vertebrae			X	X	X	X	X
Soft tissue caudal area	X	X	X	X			

Evaluating person: _______________ **Date of evaluation:** _______________

Crossed boxes: respective injury types do not apply to respective part of the body.

Chief investigator: Prof. Dr. J. Geist

Designated veterinarian: Dr. H. Kliem
